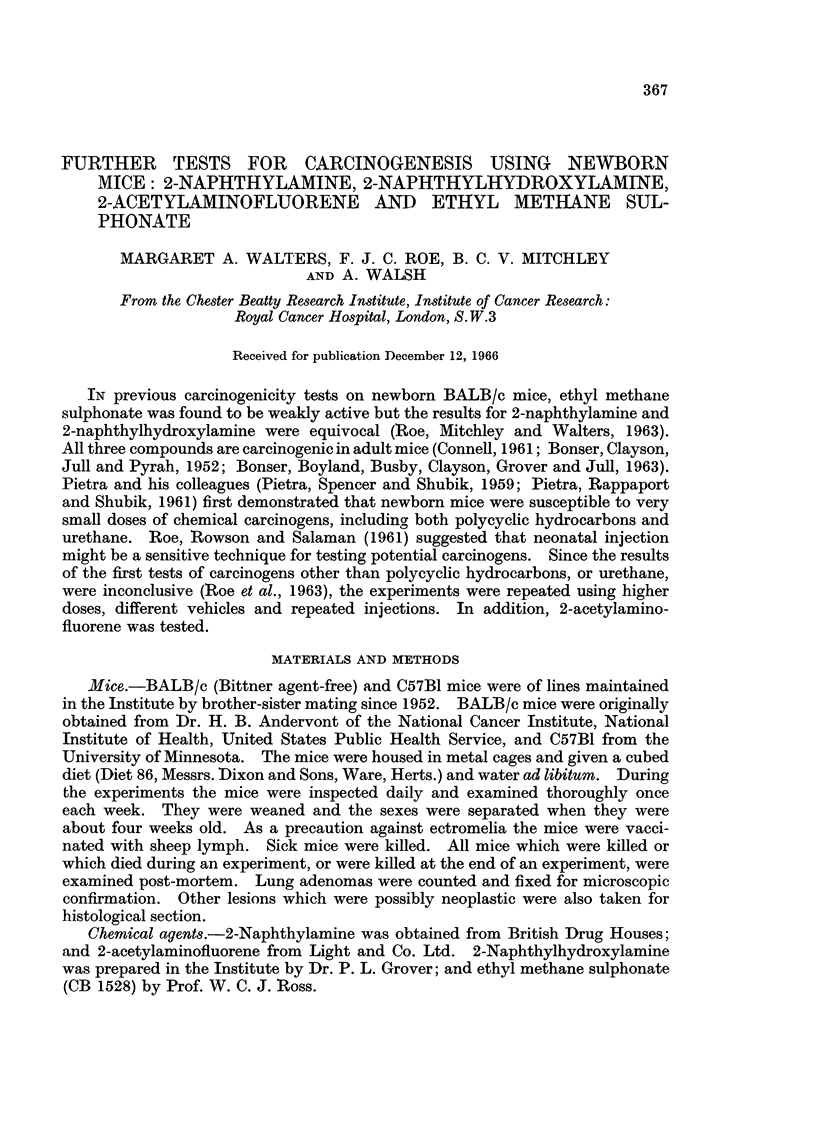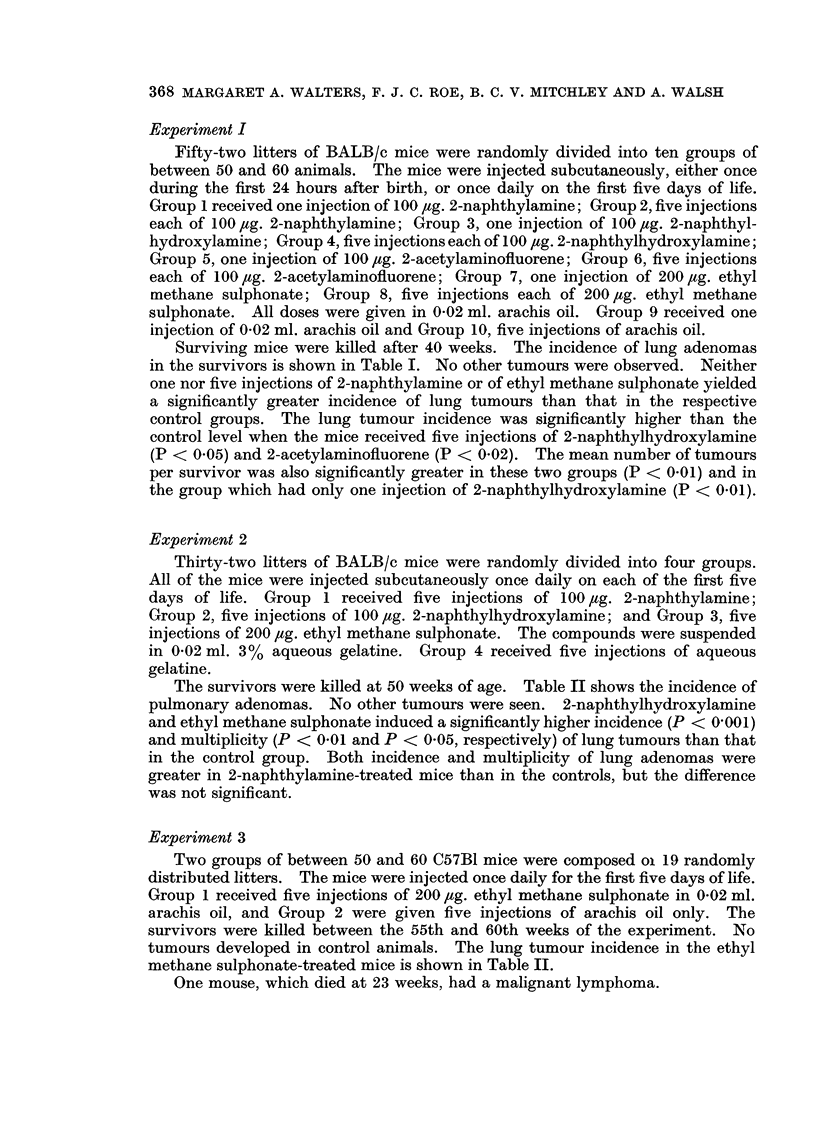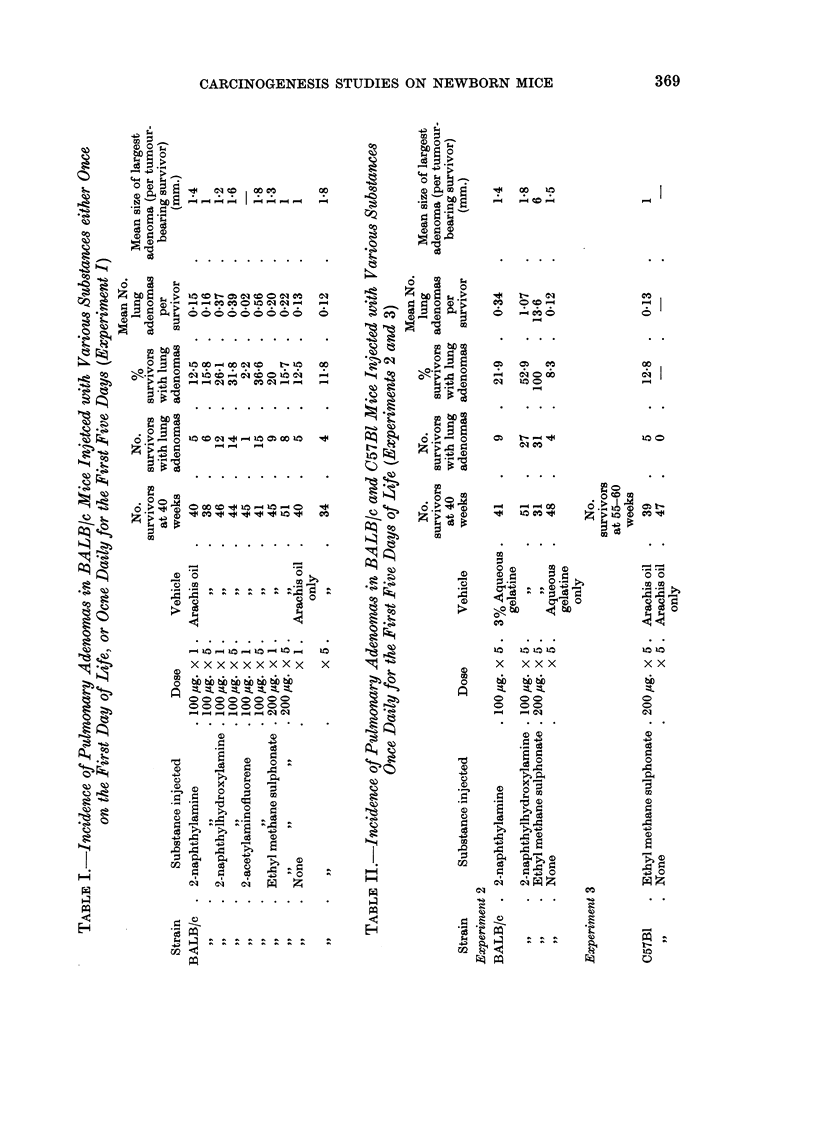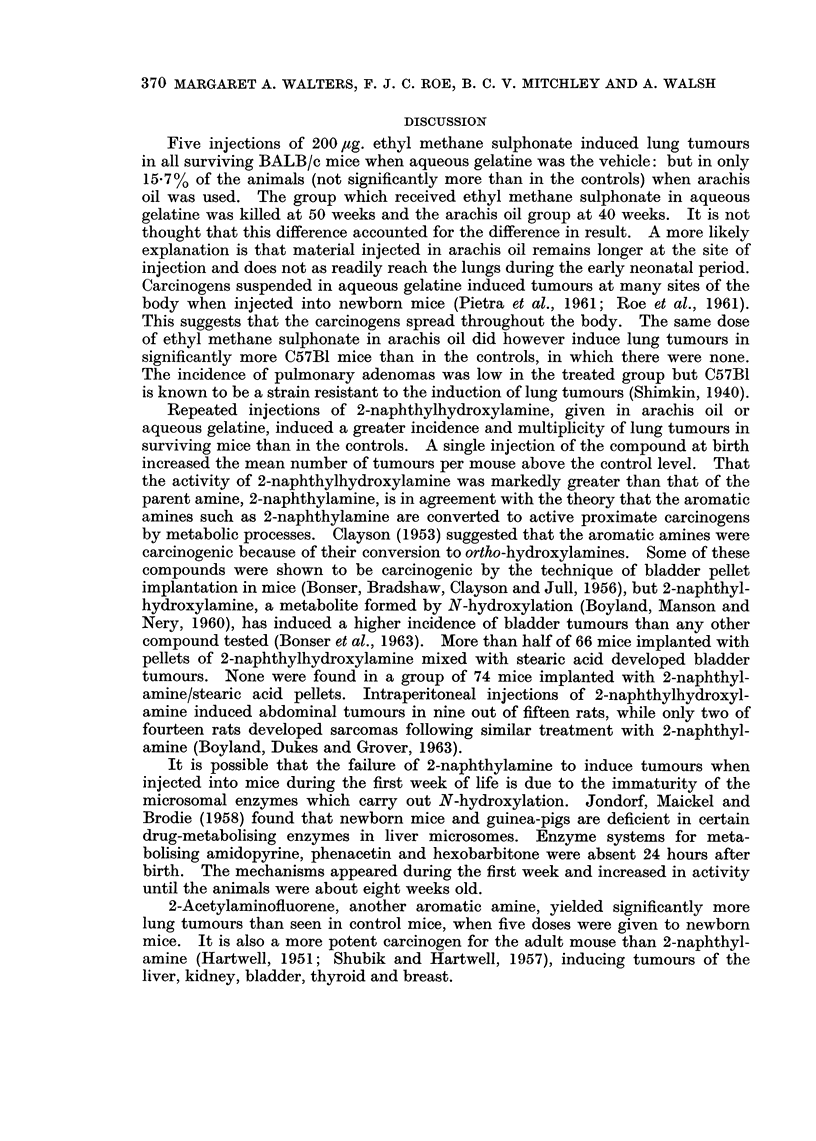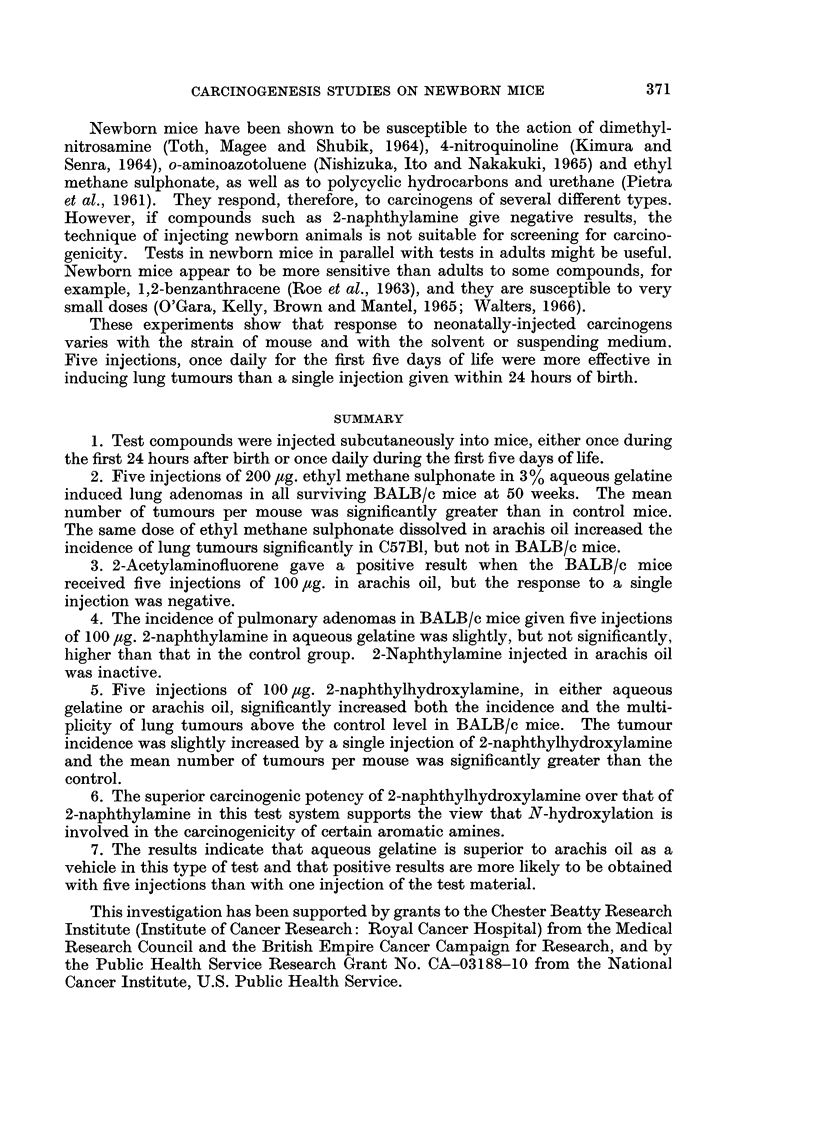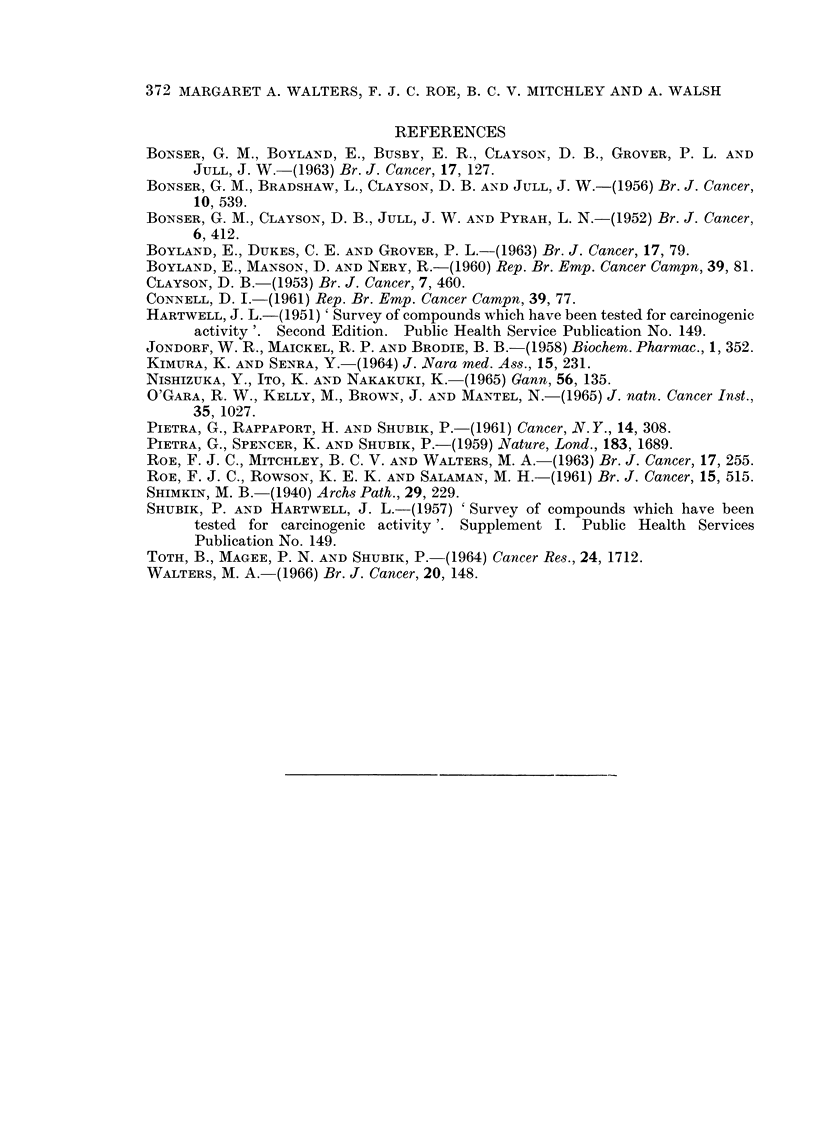# Further tests for carcinogenesis using newborn mice: 2-naphthylamine, 2-naphthylhydroxylamine, 2-acetylaminofluorene and ethyl methane sulphonate.

**DOI:** 10.1038/bjc.1967.42

**Published:** 1967-06

**Authors:** M. A. Walters, F. J. Roe, B. C. Mitchley, A. Walsh


### FURTHER TESTS FOR CARCINOGENESIS USING NEWBORN MICE: 2-NAPHTHYLAMINE, 2-NAPHTHYLHYDROXYLAMINE, 2-ACETYLAMINOFLUORENE AND ETHYL METHANE SULPHONATE

MARGARET A. WALTERS, F. J. C. ROE, B. C. V. MITCHLEY AND A. WALSH

*From the Chester Beatty Research Institute, Institute of Cancer Research: Royal Cancer Hospital, London, S.W.3*

Received for publication December 12, 1966

IN previous carcinogenicity tests on newborn BALB/c mice, ethyl methane sulphonate was found to be weakly active but the results for 2-naphthylamine and 2-naphthylhydroxylamine were equivocal (Roe, Mitchley and Walters, 1963). All three compounds are carcinogenic in adult mice (Connell, 1961; Bonser, Clayson, Jull and Pyrah, 1952; Bonser, Boyland, Busby, Clayson, Grover and Jull, 1963). Pietra and his colleagues (Pietra, Spencer and Shubik, 1959; Pietra, Rappaport and Shubik, 1961) first demonstrated that newborn mice were susceptible to very small doses of chemical carcinogens, including both polycyclic hydrocarbons and urethane. Roe, Rowson and Salaman (1961) suggested that neonatal injection might be a sensitive technique for testing potential carcinogens. Since the results of the first tests of carcinogens other than polycyclic hydrocarbons, or urethane, were inconclusive (Roe *et al.*, 1963), the experiments were repeated using higher doses, different vehicles and repeated injections. In addition, 2-acetylamino-fluorene was tested.

### MATERIALS AND METHODS

*Mice.*—BALB/c (Bittner agent-free) and C57Bl mice were of lines maintained in the Institute by brother-sister mating since 1952. BALB/c mice were originally obtained from Dr. H. B. Andervont of the National Cancer Institute, National Institute of Health, United States Public Health Service, and C57Bl from the University of Minnesota. The mice were housed in metal cages and given a cubed diet (Diet 86, Messrs. Dixon and Sons, Ware, Herts.) and water *ad libitum*. During the experiments the mice were inspected daily and examined thoroughly once each week. They were weaned and the sexes were separated when they were about four weeks old. As a precaution against ectromelia the mice were vaccinated with sheep lymph. Sick mice were killed. All mice which were killed or which died during an experiment, or were killed at the end of an experiment, were examined post-mortem. Lung adenomas were counted and fixed for microscopic confirmation. Other lesions which were possibly neoplastic were also taken for histological section.

*Chemical agents.*—2-Naphthylamine was obtained from British Drug Houses; and 2-acetylaminofluorene from Light and Co. Ltd. 2-Naphthylhydroxylamine was prepared in the Institute by Dr. P. L. Grover; and ethyl methane sulphonate (CB 1528) by Prof. W. C. J. Ross.

*Experiment I*

Fifty-two litters of BALB/c mice were randomly divided into ten groups of between 50 and 60 animals. The mice were injected subcutaneously, either once during the first 24 hours after birth, or once daily on the first five days of life. Group 1 received one injection of 100 $\mu$g. 2-naphthylamine; Group 2, five injections each of 100 $\mu$g. 2-naphthylamine; Group 3, one injection of 100 $\mu$g. 2-naphthylhydroxylamine; Group 4, five injections each of 100 $\mu$g. 2-naphthylhydroxylamine; Group 5, one injection of 100 $\mu$g. 2-acetylaminofluorene; Group 6, five injections each of 100 $\mu$g. 2-acetylaminofluorene; Group 7, one injection of 200 $\mu$g. ethyl methane sulphonate; Group 8, five injections each of 200 $\mu$g. ethyl methane sulphonate. All doses were given in 0·02 ml. arachis oil. Group 9 received one injection of 0·02 ml. arachis oil and Group 10, five injections of arachis oil.

Surviving mice were killed after 40 weeks. The incidence of lung adenomas in the survivors is shown in Table I. No other tumours were observed. Neither one nor five injections of 2-naphthylamine or of ethyl methane sulphonate yielded a significantly greater incidence of lung tumours than that in the respective control groups. The lung tumour incidence was significantly higher than the control level when the mice received five injections of 2-naphthylhydroxylamine ($P < 0·05$) and 2-acetylaminofluorene ($P < 0·02$). The mean number of tumours per survivor was also significantly greater in these two groups ($P < 0·01$) and in the group which had only one injection of 2-naphthylhydroxylamine ($P < 0·01$).

*Experiment 2*

Thirty-two litters of BALB/c mice were randomly divided into four groups. All of the mice were injected subcutaneously once daily on each of the first five days of life. Group 1 received five injections of 100 $\mu$g. 2-naphthylamine; Group 2, five injections of 100 $\mu$g. 2-naphthylhydroxylamine; and Group 3, five injections of 200 $\mu$g. ethyl methane sulphonate. The compounds were suspended in 0·02 ml. 3% aqueous gelatine. Group 4 received five injections of aqueous gelatine.

The survivors were killed at 50 weeks of age. Table II shows the incidence of pulmonary adenomas. No other tumours were seen. 2-naphthylhydroxylamine and ethyl methane sulphonate induced a significantly higher incidence ($P < 0·001$) and multiplicity ($P < 0·01$ and $P < 0·05$, respectively) of lung tumours than that in the control group. Both incidence and multiplicity of lung adenomas were greater in 2-naphthylamine-treated mice than in the controls, but the difference was not significant.

*Experiment 3*

Two groups of between 50 and 60 C57Bl mice were composed oı 19 randomly distributed litters. The mice were injected once daily for the first five days of life. Group 1 received five injections of 200 $\mu$g. ethyl methane sulphonate in 0·02 ml. arachis oil, and Group 2 were given five injections of arachis oil only. The survivors were killed between the 55th and 60th weeks of the experiment. No tumours developed in control animals. The lung tumour incidence in the ethyl methane sulphonate-treated mice is shown in Table II.

One mouse, which died at 23 weeks, had a malignant lymphoma.

TABLE I.—*Incidence of Pulmonary Adenomas in BALB/c Mice Injetced with Various Substances either Once on the First Day of Life, or Ocne Daily for the First Five Days (Experiment I)*

| Strain | Substance injected | Dose | Vehicle | No. survivors at 40 weeks | No. survivors with lung adenomas | % survivors with lung adenomas | Mean No. lung adenomas per survivor | Mean size of largest adenoma (per tumour-bearing survivor) (mm.) |
|---|---|---|---|---|---|---|---|---|
| BALB/c | 2-naphthylamine | 100 μg. × 1 | Arachis oil | 40 | 5 | 12·5 | 0·15 | 1·4 |
| ,, | ,, | 100 μg. × 5 | ,, | 38 | 6 | 15·8 | 0·16 | 1 |
| ,, | 2-naphthylhydroxylamine | 100 μg. × 1 | ,, | 46 | 12 | 26·1 | 0·37 | 1·2 |
| ,, | ,, | 100 μg. × 5 | ,, | 44 | 14 | 31·8 | 0·39 | 1·6 |
| ,, | 2-acetylaminofluorene | 100 μg. × 1 | ,, | 45 | 1 | 2·2 | 0·02 | — |
| ,, | ,, | 100 μg. × 5 | ,, | 41 | 15 | 36·6 | 0·56 | 1·8 |
| ,, | Ethyl methane sulphonate | 200 μg. × 1 | ,, | 45 | 9 | 20 | 0·20 | 1·3 |
| ,, | ,, ,, ,, | 200 μg. × 5 | ,, | 51 | 8 | 15·7 | 0·22 | 1 |
| ,, | None | × 1 | Arachis oil only | 40 | 5 | 12·5 | 0·13 | 1 |
| ,, | ,, | × 5 | ,, | 34 | 4 | 11·8 | 0·12 | 1·8 |

TABLE II.—*Incidence of Pulmonary Adenomas in BALB/c and C57Bl Mice Injected with Various Substances Once Daily for the First Five Days of Life (Experiments 2 and 3)*

| Strain | Substance injected | Dose | Vehicle | No. survivors at 40 weeks | No. survivors with lung adenomas | % survivors with lung adenomas | Mean No. lung adenomas per survivor | Mean size of largest adenoma (per tumour-bearing survivor) (mm.) |
|---|---|---|---|---|---|---|---|---|
| *Experiment* 2 | | | | | | | | |
| BALB/c | 2-naphthylamine | 100 μg. × 5 | 3% Aqueous gelatine | 41 | 9 | 21·9 | 0·34 | 1·4 |
| ,, | 2-naphthylhydroxylamine | 100 μg. × 5 | ,, | 51 | 27 | 52·9 | 1·07 | 1·8 |
| ,, | Ethyl methane sulphonate | 200 μg. × 5 | ,, | 31 | 31 | 100 | 13·6 | 6 |
| ,, | None | × 5 | Aqueous gelatine only | 48 | 4 | 8·3 | 0·12 | 1·5 |
| *Experiment* 3 | | | | No. survivors at 55–60 weeks | | | | |
| C57Bl | Ethyl methane sulphonate | 200 μg. × 5 | Arachis oil | 39 | 5 | 12·8 | 0·13 | 1 |
| ,, | None | × 5 | Arachis oil only | 47 | 0 | — | — | — |

### DISCUSSION

Five injections of 200 $\mu$g. ethyl methane sulphonate induced lung tumours in all surviving BALB/c mice when aqueous gelatine was the vehicle: but in only 15·7% of the animals (not significantly more than in the controls) when arachis oil was used. The group which received ethyl methane sulphonate in aqueous gelatine was killed at 50 weeks and the arachis oil group at 40 weeks. It is not thought that this difference accounted for the difference in result. A more likely explanation is that material injected in arachis oil remains longer at the site of injection and does not as readily reach the lungs during the early neonatal period. Carcinogens suspended in aqueous gelatine induced tumours at many sites of the body when injected into newborn mice (Pietra *et al.*, 1961; Roe *et al.*, 1961). This suggests that the carcinogens spread throughout the body. The same dose of ethyl methane sulphonate in arachis oil did however induce lung tumours in significantly more C57Bl mice than in the controls, in which there were none. The incidence of pulmonary adenomas was low in the treated group but C57Bl is known to be a strain resistant to the induction of lung tumours (Shimkin, 1940).

Repeated injections of 2-naphthylhydroxylamine, given in arachis oil or aqueous gelatine, induced a greater incidence and multiplicity of lung tumours in surviving mice than in the controls. A single injection of the compound at birth increased the mean number of tumours per mouse above the control level. That the activity of 2-naphthylhydroxylamine was markedly greater than that of the parent amine, 2-naphthylamine, is in agreement with the theory that the aromatic amines such as 2-naphthylamine are converted to active proximate carcinogens by metabolic processes. Clayson (1953) suggested that the aromatic amines were carcinogenic because of their conversion to *ortho*-hydroxylamines. Some of these compounds were shown to be carcinogenic by the technique of bladder pellet implantation in mice (Bonser, Bradshaw, Clayson and Jull, 1956), but 2-naphthylhydroxylamine, a metabolite formed by *N*-hydroxylation (Boyland, Manson and Nery, 1960), has induced a higher incidence of bladder tumours than any other compound tested (Bonser *et al.*, 1963). More than half of 66 mice implanted with pellets of 2-naphthylhydroxylamine mixed with stearic acid developed bladder tumours. None were found in a group of 74 mice implanted with 2-naphthylamine/stearic acid pellets. Intraperitoneal injections of 2-naphthylhydroxylamine induced abdominal tumours in nine out of fifteen rats, while only two of fourteen rats developed sarcomas following similar treatment with 2-naphthylamine (Boyland, Dukes and Grover, 1963).

It is possible that the failure of 2-naphthylamine to induce tumours when injected into mice during the first week of life is due to the immaturity of the microsomal enzymes which carry out *N*-hydroxylation. Jondorf, Maickel and Brodie (1958) found that newborn mice and guinea-pigs are deficient in certain drug-metabolising enzymes in liver microsomes. Enzyme systems for metabolising amidopyrine, phenacetin and hexobarbitone were absent 24 hours after birth. The mechanisms appeared during the first week and increased in activity until the animals were about eight weeks old.

2-Acetylaminofluorene, another aromatic amine, yielded significantly more lung tumours than seen in control mice, when five doses were given to newborn mice. It is also a more potent carcinogen for the adult mouse than 2-naphthylamine (Hartwell, 1951; Shubik and Hartwell, 1957), inducing tumours of the liver, kidney, bladder, thyroid and breast.

Newborn mice have been shown to be susceptible to the action of dimethylnitrosamine (Toth, Magee and Shubik, 1964), 4-nitroquinoline (Kimura and Senra, 1964), *o*-aminoazotoluene (Nishizuka, Ito and Nakakuki, 1965) and ethyl methane sulphonate, as well as to polycyclic hydrocarbons and urethane (Pietra *et al.*, 1961). They respond, therefore, to carcinogens of several different types. However, if compounds such as 2-naphthylamine give negative results, the technique of injecting newborn animals is not suitable for screening for carcinogenicity. Tests in newborn mice in parallel with tests in adults might be useful. Newborn mice appear to be more sensitive than adults to some compounds, for example, 1,2-benzanthracene (Roe *et al.*, 1963), and they are susceptible to very small doses (O'Gara, Kelly, Brown and Mantel, 1965; Walters, 1966).

These experiments show that response to neonatally-injected carcinogens varies with the strain of mouse and with the solvent or suspending medium. Five injections, once daily for the first five days of life were more effective in inducing lung tumours than a single injection given within 24 hours of birth.

### SUMMARY

1. Test compounds were injected subcutaneously into mice, either once during the first 24 hours after birth or once daily during the first five days of life.

2. Five injections of 200 μg. ethyl methane sulphonate in 3% aqueous gelatine induced lung adenomas in all surviving BALB/c mice at 50 weeks. The mean number of tumours per mouse was significantly greater than in control mice. The same dose of ethyl methane sulphonate dissolved in arachis oil increased the incidence of lung tumours significantly in C57Bl, but not in BALB/c mice.

3. 2-Acetylaminofluorene gave a positive result when the BALB/c mice received five injections of 100 μg. in arachis oil, but the response to a single injection was negative.

4. The incidence of pulmonary adenomas in BALB/c mice given five injections of 100 μg. 2-naphthylamine in aqueous gelatine was slightly, but not significantly, higher than that in the control group. 2-Naphthylamine injected in arachis oil was inactive.

5. Five injections of 100 μg. 2-naphthylhydroxylamine, in either aqueous gelatine or arachis oil, significantly increased both the incidence and the multiplicity of lung tumours above the control level in BALB/c mice. The tumour incidence was slightly increased by a single injection of 2-naphthylhydroxylamine and the mean number of tumours per mouse was significantly greater than the control.

6. The superior carcinogenic potency of 2-naphthylhydroxylamine over that of 2-naphthylamine in this test system supports the view that *N*-hydroxylation is involved in the carcinogenicity of certain aromatic amines.

7. The results indicate that aqueous gelatine is superior to arachis oil as a vehicle in this type of test and that positive results are more likely to be obtained with five injections than with one injection of the test material.

This investigation has been supported by grants to the Chester Beatty Research Institute (Institute of Cancer Research: Royal Cancer Hospital) from the Medical Research Council and the British Empire Cancer Campaign for Research, and by the Public Health Service Research Grant No. CA–03188–10 from the National Cancer Institute, U.S. Public Health Service.

### REFERENCES

BONSER, G. M., BOYLAND, E., BUSBY, E. R., CLAYSON, D. B., GROVER, P. L. AND JULL, J. W.—(1963) *Br. J. Cancer*, **17**, 127.

BONSER, G. M., BRADSHAW, L., CLAYSON, D. B. AND JULL, J. W.—(1956) *Br. J. Cancer*, **10**, 539.

BONSER, G. M., CLAYSON, D. B., JULL, J. W. AND PYRAH, L. N.—(1952) *Br. J. Cancer*, **6**, 412.

BOYLAND, E., DUKES, C. E. AND GROVER, P. L.—(1963) *Br. J. Cancer*, **17**, 79.

BOYLAND, E., MANSON, D. AND NERY, R.—(1960) *Rep. Br. Emp. Cancer Campn*, **39**, 81.

CLAYSON, D. B.—(1953) *Br. J. Cancer*, **7**, 460.

CONNELL, D. I.—(1961) *Rep. Br. Emp. Cancer Campn*, **39**, 77.

HARTWELL, J. L.—(1951) 'Survey of compounds which have been tested for carcinogenic activity'. Second Edition. Public Health Service Publication No. 149.

JONDORF, W. R., MAICKEL, R. P. AND BRODIE, B. B.—(1958) *Biochem. Pharmac.*, **1**, 352.

KIMURA, K. AND SENRA, Y.—(1964) *J. Nara med. Ass.*, **15**, 231.

NISHIZUKA, Y., ITO, K. AND NAKAKUKI, K.—(1965) *Gann*, **56**, 135.

O'GARA, R. W., KELLY, M., BROWN, J. AND MANTEL, N.—(1965) *J. natn. Cancer Inst.*, **35**, 1027.

PIETRA, G., RAPPAPORT, H. AND SHUBIK, P.—(1961) *Cancer, N.Y.*, **14**, 308.

PIETRA, G., SPENCER, K. AND SHUBIK, P.—(1959) *Nature, Lond.*, **183**, 1689.

ROE, F. J. C., MITCHLEY, B. C. V. AND WALTERS, M. A.—(1963) *Br. J. Cancer*, **17**, 255.

ROE, F. J. C., ROWSON, K. E. K. AND SALAMAN, M. H.—(1961) *Br. J. Cancer*, **15**, 515.

SHIMKIN, M. B.—(1940) *Archs Path.*, **29**, 229.

SHUBIK, P. AND HARTWELL, J. L.—(1957) 'Survey of compounds which have been tested for carcinogenic activity'. Supplement I. Public Health Services Publication No. 149.

TOTH, B., MAGEE, P. N. AND SHUBIK, P.—(1964) *Cancer Res.*, **24**, 1712.

WALTERS, M. A.—(1966) *Br. J. Cancer*, **20**, 148.